# Domestication leads to increased predation susceptibility

**DOI:** 10.1038/s41598-020-58661-9

**Published:** 2020-02-06

**Authors:** Monica F. Solberg, Grethe Robertsen, Line E. Sundt-Hansen, Kjetil Hindar, Kevin A. Glover

**Affiliations:** 1 Institute of Marine Research, P.O. Box 1870 Nordnes, NO, 5817 Bergen, Norway; 2 Norwegian Institute for Nature Research (NINA), P.O. Box 5685 Torgarden, NO, 7485 Trondheim, Norway; 3 Department of Biology, University of Bergen, Bergen, Norway

**Keywords:** Experimental evolution, Animal breeding, Genetic hybridization, Conservation biology

## Abstract

Domestication involves adapting animals to the human-controlled environment. Genetic changes occurring during the domestication process may manifest themselves in phenotypes that render domesticated animals maladaptive for life in the wild. Domesticated Atlantic salmon frequently interbreed with wild conspecifics, and their offspring display reduced survival in the wild. However, the mechanism(s) contributing to their lower survival in the wild remains a subject of conjecture. Here, we document higher susceptibility to predation by brown trout in fast-growing domesticated salmon, as compared to their slow-growing wild conspecifics, demonstrating that directional selection for increased growth comes at a cost of decreased survival when under the risk of predation, as predicted by the growth/predation risk trade-off. Despite earlier documentation of altered risk-taking behavior, this study demonstrates for the first time that domestication of Atlantic salmon has lead to increased predation susceptibility, and that this consitutes a mechanism underpinning the observed survial differences in the wild.

## Introduction

Domestication, the process by which animals become adapted to the human-controlled environment^1,2^, has been used to tame a wide variety of species for the benefit of humans^3^. Classic examples include livestock such as sheep, pigs, poultry and cattle raised for food production^4–7^, dogs and horses for human companionship and assistance^8,9^, and fish and birds for aesthetic pleasure^10^. A common feature displayed is that selection for desirable traits has resulted in significant genetic and phenotypic changes over time^11,12^. However, relaxation of natural selection has permitted, and in some cases perpetuated, the development of new and sometimes striking phenotypes that probably did not exist in the wild^8,10^.

The process of domestication, which changes the genetic make-up of species being reared for human benefit, has not only resulted in beneficial changes. Some of the phenotypic traits deliberately selected for, or inadvertently accumulated during domestication, render the animals themselves unlikely to be fit for a life in the wild, if they were to accidently escape or to be deliberately released. For example, some livestock breeds now display reproductive problems without human interference^13^, developmental and growth issues^14,15^ and behavioral abnormalities^2,16^. Reduced survival of domesticated animals in the wild has also been documented^17–21^, although the underlying mechanisms are seldom resolved.

Atlantic salmon (*Salmo salar* L.) is regarded as one of the most domesticated fish species reared for food^22^, although its history of domestication is short compared to traditional livestock which have been under domestication for 1000 s of year in some cases^10^. Domestication of Atlantic salmon started in the early 1970’s by capturing adult fish from a number of rivers and taking them into breeding programs^23,24^. Thereafter, directional selection for important traits, including rapid growth and delayed maturation was conducted through phenotypic and family-based selection^24,25^. As a consequence, both economically important traits and traits that have not been under directional selection have been significantly changed^26^. Although domesticated salmon have not been altered to the extent that they bear little resemblance to their wild ancestors (as is the case for several domesticated animals), genetic differences in a wide range of phenotypic traits now exist between wild and domesticated conspecifics^27^. The most striking of these changes is growth, whereby the offspring of farmed salmon now out-grow the offspring of wild salmon several-fold when reared communally under farming conditions, both during the juvenile^26,28,29^ and the adult stage^30^.

For Atlantic salmon, one of the major domestication-driven changes that has been revealed is the decreased ability of their offspring to survive in the wild^17–21^, which may reduce productivity in populations following introgression^17,31^. This is of major concern given that in the past 3–4 decades, tens of millions of domesticated salmon have escaped into the wild, and as a result, introgression is now observed in a large number of wild populations^32–40^. Several studies have attempted to identify the causative mechanisms for the observed difference in survival between the offspring of domesticated and wild Atlantic salmon in the natural environment, and a difference in predation susceptibility has been suggested as a potential explanatory factor^41–43^. Due to a trade-off between growth and survival in the wild^44,45^, the deviating growth potential of domesticated and wild salmon is likely to have resulted in correspondingly deviating risks of predation^46^. Behavioral studies have indeed indicated that a lack of predator awareness and/or increased risk taking behavior may be of relevance^47–49^, especially given the fact that predation is considered a major mortality factor of salmonid juveniles in the wild^50,51^. However, thus far, no study has been able to unequivocally document whether fish-predation underpins survival differences between offspring of domesticated and wild salmon in the natural environment^41,42^.

**Figure 1:**
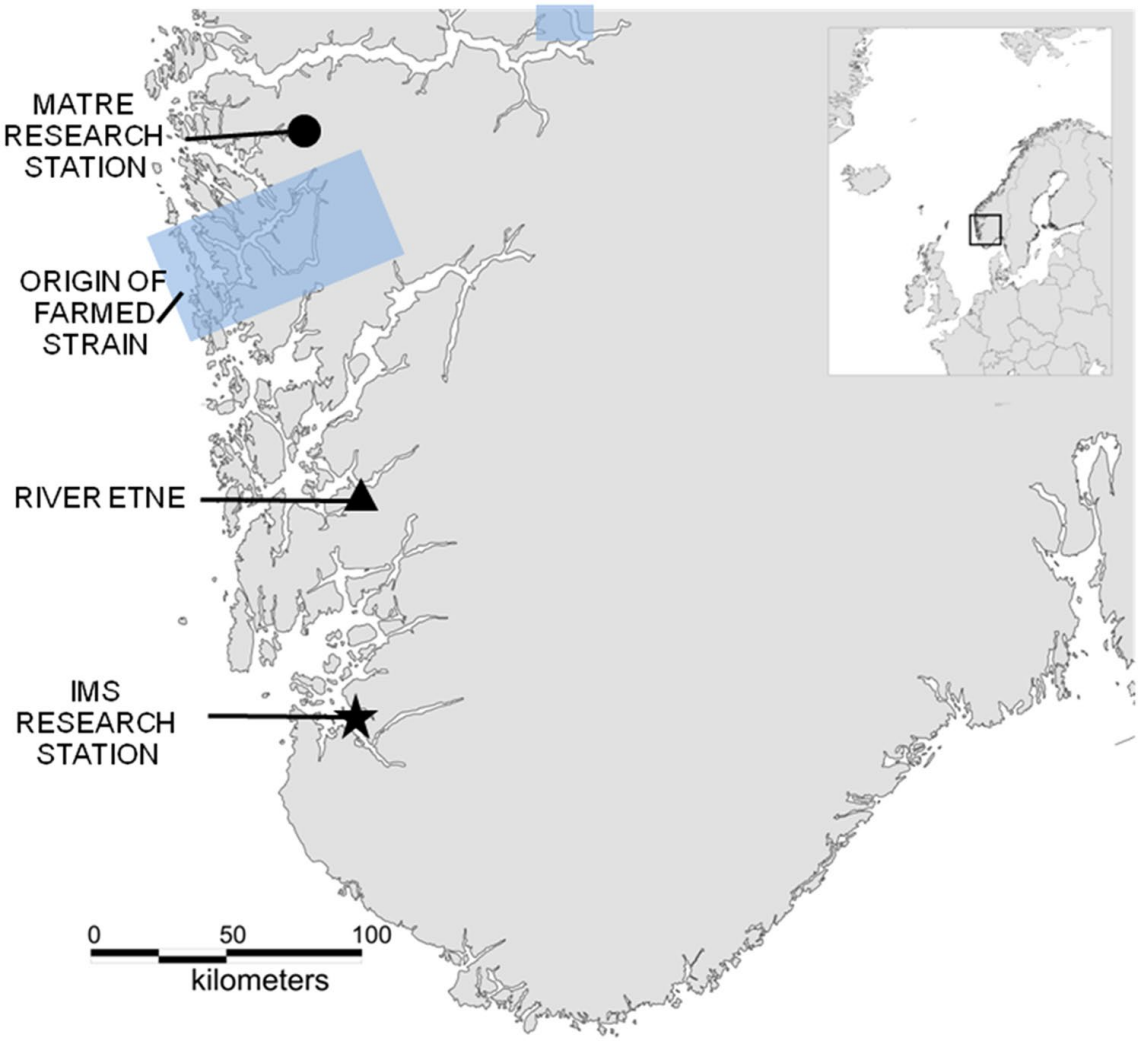
Map. Study area showing the origin of the domesticated (farmed) strain and the location of the wild strain (river Etne, 59°40′N, 5°56′E) from which gametes were collected and brought to Matre Research station (IMR) where the experimental domesticated, wild and F1 hybrid crosses were produced. Eyed eggs were sent to the NINA Research Station at Ims, where the experiment was conducted during the early juvenile stage.

Atlantic salmon provides a good model in which to study the mechanisms underlying reduced survival of domesticated fish in the wild. This is due to several key attributes including its degree of domestication, magnitude of interbreeding between domesticated and wild conspecifics, and status of knowledge^27^. Here, we investigate whether the growth/predation risk trade-off can explain why offspring of domesticated salmon display reduced survival in the wild compared to offspring of their wild conspecifics. Growth and survival of domesticated, wild and F1 hybrid offspring (Fig. 1) was studied from the onset of start feeding along an environmental gradient: i) standard hatchery conditions with a surplus of feed, ii) a semi-natural environment with competition for restricted natural feed, iii) a semi-natural environment with competition for restricted natural feed under the risk of predation by brown trout (*Salmo trutta* L.). Six weeks after onset of the experiment, surviving juveniles were recaptured and identified to origin by the use of DNA. The stomach contents of all trout were sequenced, specifically searching for salmon DNA to confirm predation.

## Results

### Initial weight

**Figure 2:**
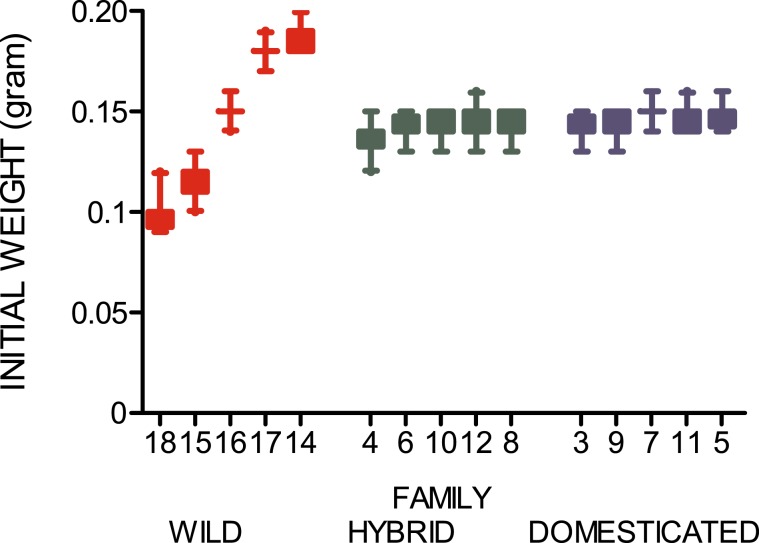
Initial weights of all genetic groups. Observed body mass of wild, F1 hybrid and domesticated Atlantic salmon families prior to the onset of the experiment (yolk-sac fry). Numbers on the x-axis represent the identity of the different families. Colours illustrate the strains (wild = red, F1 hybrid = green or domesticated = blue). Error bars show the 5–95 percentile (total n = 300).

At the start of the experiment, no difference in weight was detected between yolk-sac fry of domesticated, hybrid and wild origin (LME: genetic origin: F_2, 5_ = 3.4, P = 0.1, domesticated yolk-sac fry 0.146 ± 0.007 g, hybrid yolk-sac fry 0.142 ± 0.007 g and wild yolk-sac fry 0.146 ± 0.035 g; mean ± SD). However, variation in initial weight due to maternal (S.D. = 0.025, P < 0.001) and paternal (S.D. = 0.003, P = 0.07) origin was detected among the three groups, Fig. 2.

### Growth

**Figure 3:**
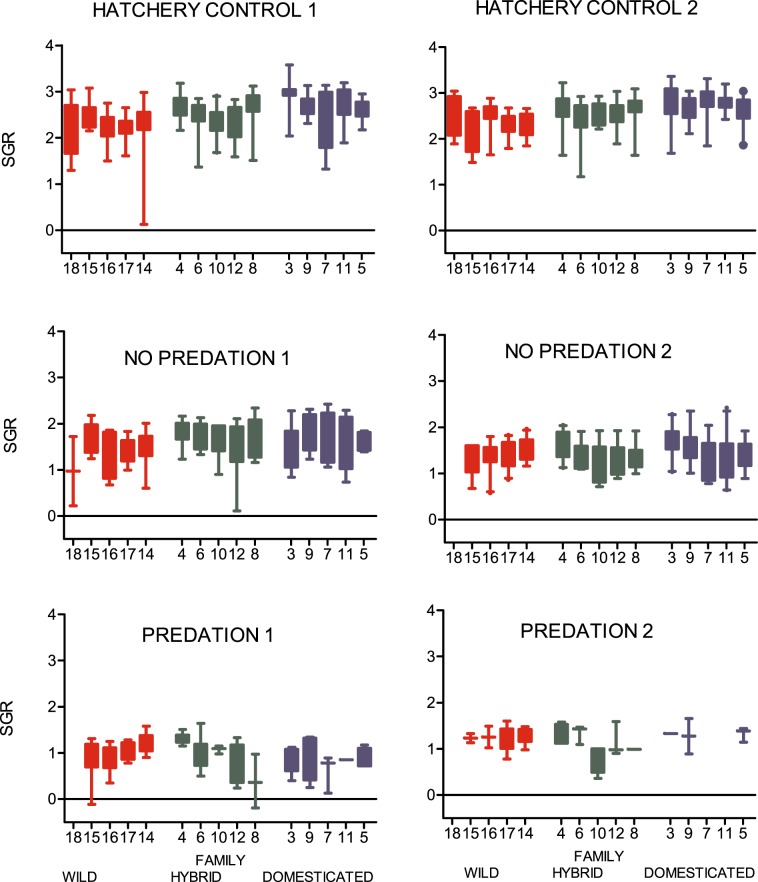
Body mass at termination of all genetic groups. Specific growth rate (SGR) of wild, F1 hybrid and domesticated Atlantic salmon families in the indoor hatchery tanks with surplus of feed (hatchery control), in the outdoor semi-natural stream channels with competition for natural feed (no predation) and in the semi-natural stream channels with competition for natural feed under the risk of trout predation (predation). Numbers on the x-axis represent the identities of the different families. Colours illustrate the strains (wild = red, F1 hybrid = green or domesticated = blue). Error bars show the 5–95 percentile (total n = 512, 368 and 147 in the hatchery controls, the no predation treatment and the predation treatment, respectively).

Upon termination, fry in the hatchery tanks fed ad libitum had in general grown more than fry in the semi-natural stream channels while only small differences in growth rate were detected between fry in the semi-natural stream channels with or without predation (Linear mixed effect model (LME):treatment: F_2, 3_ = 107, P = 0.002; *post hoc* Tukey: hatchery versus no predation, P < 0.006; hatchery versus predation, P < 0.001; no predation versus predation, P < 0.04; hatchery 2.5 ± 0.07 SGR, no predation 1.5 ± 0.07 SGR and predation 1.07 ± 0.08 SGR; estimated mean ± SE; Fig. 3).

Differences in growth rate were detected between fry of domesticated, hybrid and wild origin in the hatchery, but not in the semi-natural stream channels both with or without predation (LME: genetic origin by environment interaction: F_4, 1000_ = 10.3, P < 0.001; genetic origin: F_2, 17 = _2.6, P = 0.1). Domesticated fry grew significantly faster than both the hybrid and the wild salmon in the hatchery tanks (*post hoc* Tukey: domesticated versus hybrid, P = 0.01; domesticated versus wild, P < 0.001; domesticated fry 2.7 ± 0.08 SGR, hybrid fry 2.5 ± 0.08 SGR and wild fry 2.3 ± 0.08 SGR; estimated mean ± SE; Fig. 3). Hybrid salmon grew also significantly faster than the wild salmon in this environment (*post hoc* Tukey, P = 0.005; Fig. 3). However, in the semi-natural stream channels with restricted natural feed without predation, salmon of all genetic groups grew at a similar rate (*post hoc* Tukey: no predation; domesticated versus hybrid, P = 0.9; domesticated versus wild, P = 0.2, hybrid versus wild, P = 0.3; domesticated fry 1.5 ± 0.08 SGR, hybrid fry 1.5 ± 0.08 SGR and wild fry 1.4 ± 0.08 SGR; estimated mean ± SE, Fig. 3). In the semi-natural stream channels with both restricted natural feed and predation, growth rate was also similar for salmon of all genetic groups (*post hoc* Tukey: predation; domesticated versus hybrid, P = 0.5; domesticated versus wild, P = 0.3, hybrid versus wild, P = 0.9; domesticated fry 1.0 ± 0.11 SGR, hybrid fry 1.1 ± 0.09 SGR and wild fry 1.1 ± 0.09 SGR; estimated mean ± SE, Fig. 3).

Variation in growth rate due to maternal identity (S.D. = 0.05, P = 0.09), paternal identity (S.D. = 0.06, P = 0.04) and replicate (S.D. = 0.09, P = 0.0001) was detected and controlled for as random factors in the linear mixed effect model.

### Survival

**Figure 4:**
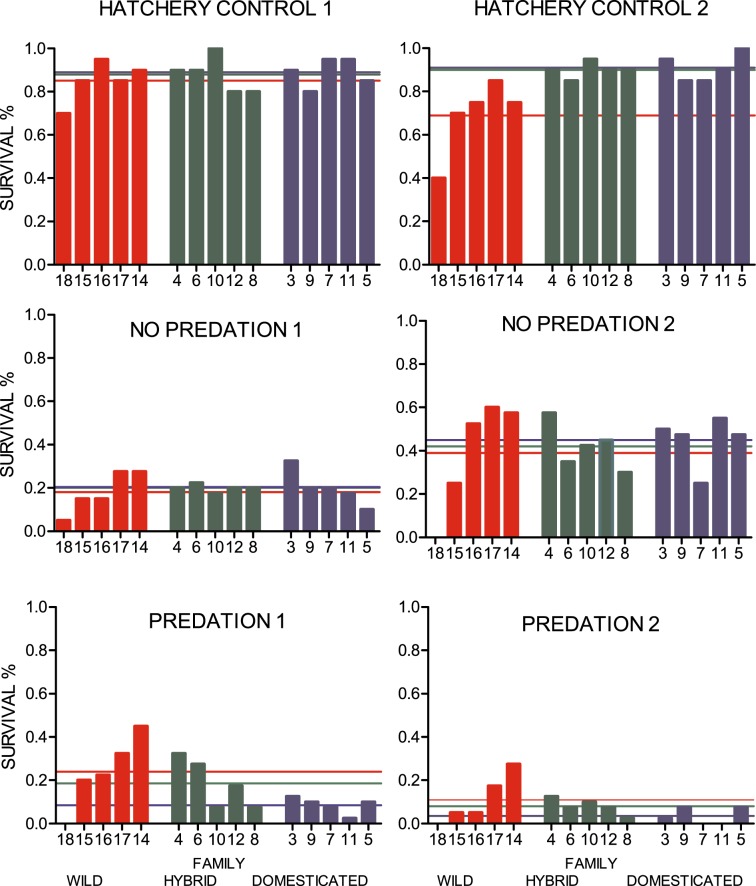
Observed survival of all genetic groups. Observed survival (%) of wild, F1 hybrid and domesticated Atlantic salmon families in the indoor hatchery tanks with surplus of feed (hatchery control), in the outdoor semi-natural stream channels with competition for natural feed (no predation) and in the semi-natural stream channels with competition for natural feed under the risk of trout predation (predation). Numbers on the x-axis represents the identity of the different families. Coloured lines illustrate the strains (wild = red, F1 hybrid = green or domesticated = blue) average survival in the respective replicate (total n = 512, 368 and 147 in the hatchery controls, the no predation treatment and the predation treatment, respectively).

In the two indoor hatchery tanks with excess food and no predation, 512 of 600 fish (85.3%) were sampled at the end of the experiment and identified to family (Fig. 4). Of the 1200 individuals placed into the two semi-natural stream channels with competition for natural food but without the trout predators, 368 (30.7%) were recaptured at the end of the experiment (Fig. 4). Of the 1200 individuals placed into the two semi-natural stream channels with competition for natural food and in the presence of trout predators, 147 (12.3%) were recaptured (Fig. 4). Thus, overall survival/recapture rates were significantly higher in the indoor hatchery tanks compared to the semi-natural stream channels (*post hoc* Tukey: hatchery versus no predation, P < 0.001; hatchery versus predation, P < 0.001). In the semi-natural stream channels, survival/recapture rates were significantly higher in the absence of predation (*post hoc* Tukey: P = 0.02).

**Figure 5:**
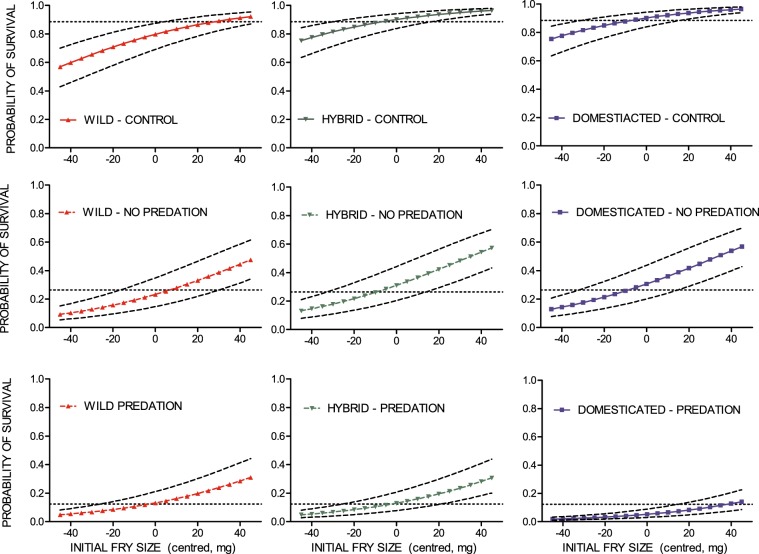
Probability of survival for all genetic groups. Estimated probability of survival, against initial fry size (centred, mg), of wild, F1 hybrid and domesticated Atlantic salmon in the indoor hatchery tanks with surplus of feed (hatchery control), in the outdoor semi-natural stream channels with competition for natural feed (no predation) and in the semi-natural channels with competition for natural feed under the risk of trout predation (predation). Dotted lines illustrate uncertainty caused by the random effect of family, maternal identity and replicate.

Differences in survival between fry of domesticated, hybrid and wild origin were detected in the hatchery and the semi-natural stream channels with predation, but not in the steam channels without predation (GLMM; environment by genetic group interaction, P < 0.001). Survival rates of domesticated and hybrid salmon were similar in the indoor hatchery tanks (*post hoc* Tukey: P = 1), both being higher than wild salmon in this environment (*post hoc* Tukey: domesticated versus wild, P = 0.046; hybrid versus wild, P = 0.04). In the semi-natural stream channels without predation, survival of all three genetic groups was similar (*post hoc* Tukey: domesticated versus hybrid, P = 1; domesticated versus wild, P = 0.3; hybrid versus wild, P = 0.3). However, in the semi-natural stream channels with trout predation, domesticated salmon had a significantly lower survival rate compared to both the wild (*post hoc* Tukey: P = 0.005) and the hybrid salmon (*post hoc* Tukey: P < 0.001). Hybrid and wild salmon displayed similar survival rates in this environment (*post hoc* Tukey: P = 1) after controlling for initial weight which had an overall positive effect on survival (GLMM: P < 0.001) (Fig. 5). Variation in survival due to family background (S.D. < 0.001), maternal identity (S.D. = 0.3) and replicate (S.D. = 0.45) were detected and controlled for as random factors in the generalised linear mixed effect model.

## Discussion

Here, we provide the first empirical evidence that the domestication of Atlantic salmon has lead to increased predation susceptibility by brown trout, and that this therefore consitutes a mechanism underpinning the observed differences in survival between domesticated and wild salmon in the natural habitat^17–21^. Domesticated Atlantic salmon frequently escape from fish farms, and interact with wild salmon in the natural habitat^27^. Therefore, our work enlightens current understanding of the way in which a domesticated species may interact with wild conspecifics in the natural environment.

Domestication involves adaptation to the human-controlled environment, and only a fraction of the species attempted have been successfully domesticated^3^. One of the main obstacles for domestication is not being able to successfully reduce the species sensitivity to the novel environment in which they are cultured^1,8^. In salmon, a reduction in environmental sensitivity^26^ and environmental information processing^52^ has evolved inadvertently, likely as a consequence of directional selection for increased growth rate^25,53^ and the relaxation of natural selection. Candidate broodfish displaying an overall reduced sensitivity to the cultured environment are likely to feed at a higher rate than candidate broodfish less tolerant to a broad range of environmental stressors, resulting in a higher growth rate and hence increased chance of being selected to propagate the next generation. This mechanism inadvertently selects for increased risk-taking behaviour^47–49^ as well as increased predation-related stress resistance^54^. Furthermore, due to the relaxation of natural selection, traits that are crucial for survival in the wild, like predator avoidance, but are costly to maintain if they provide no competitive advantage^55^, may erode in the domesticated environment in the absence of predation. Thus, a reduction in predator awareness and increased predation susceptibility may accompany successful domestication of fast-growing Atlantic salmon. In this study, similar survival rates were observed in offspring of domesticated, hybrid and wild origin in a semi-natural environment with intra-specific competition for natural feed. However, significantly lower survival rates were observed in the offspring of domesticated origin, as compared to offspring of wild origin, in an identical semi-natural environment where larger brown trout were present as predators. Therefore, although previous work has documented increased risk-taking behaviour in domesticated salmon^47–49^, our study has made an important advance by providing the first documentation of increased susceptibility to predation in domesticated salmon. In turn, this demonstrates the importance of this mechanism in the observed survival differences in the natural environment.

For salmonids, a trade-off between survival and growth has been documented in the presence of predators in the natural environment^45^. Thus, theory predicts that for offspring of domesticated salmon with a genetic potential for increased growth rate^56^, this should come at a cost of reduced life-time success. As the domesticated salmon grew faster than their wild conspecifics under hatchery conditions, while displaying lower survival rates when under the risk of predation in a semi-natural environment, we demonstrate that domesticated salmon with genetic potential of fast growth, display significantly higher mortality rates when exposed to predation, than their wild conspecifics. Competition for feed, or a reduced ability to catch live prey has also been suggested as an explanation for why offspring of farmed salmon display reduced survival in the wild^17–20^. However, as our study shows, competition for natural feed in a predator free environment, did not result in significant differences in survival between offspring of domesticated and wild origin. In fact, in the absence of predation and inter-specific competition, domesticated salmon displayed higher, although not significantly different, survival rates compared to the wild salmon. This trend supports the previous observation that offspring of domesticated salmon are good competitors under environmental conditions where feed are limited and an ignorant and bold behaviour comes with no immediate penalty^28,57^. In fact, offspring of domesticated salmon have been documented to outcompete offspring of wild origin in the absence of predation, when competing for limited resources under semi-natural conditions^43^.

The cost connected to higher growth potential, in terms of increased predation susceptibility, may explain why the severalfold elevated growth rates of domesticated and admixed salmon seen in the hatchery are modest or absent in the wild^19–21,58,59^. However, a recent study demonstrated that selection against domesticated salmon with an increased genetic growth potential in the wild does not result in complete removal of domesticated fish with elevated growth potential^56^. Plasticity towards feed availability and/or accessibility also significantly regulates the growth rate of domesticated salmon in the natural environment, resulting in more similar growth rates in salmon of all origins^19,20,56^, which we also detected in the semi-natural environments with restricted natural feed.

Atlantic salmon represents a unique example of evolution in action as a domesticated species that frequently interbreeds with its wild conspecifics^27,60^. Annually, hundreds of thousands or millions of domesticated salmon escape, many enter rivers supporting wild populations^40,61–63^, and introgression (that is sometimes extensive) has been documented in populations in Norway, Scotland, Ireland and Canada^32–40,64^. The long-term evolutionary consequences of introgression are of concern, not least because the offspring of domesticated salmon display reduced survival in the wild^17–21^, which may undermine the viability and abundance of wild populations^31^. However, the mechanisms underlying fitness differences between domesticated and wild salmon in the natural habitat has been a subject of extensive conjecture. To this end, our results provide the first evidence that domestication of Atlantic salmon has permitted evolution of increased predation susceptibility, and that predation by brown trout represents a mechanism underpinning survival differences in the wild.

## Methods

### Experimental populations

Gametes from ten wild Atlantic salmon originating from the River Etne (59°40′N, 5°56′E) and ten domesticated salmon originating from the Norwegian Mowi strain were used to generate three cross types for this experiment; (i) five pure wild families; (ii) five pure domesticated families and (iii) five wild x domesticated F1 hybrid families. Eggs of each of the five domesticated female were split in two and fertilised by one domesticated and one wild male, thus creating F1 hybrid families that were maternal and paternal half-siblings of the domesticated and wild families, respectively (see Supplementary file 1 for full overview of the half-sibling design).

Wild parental salmon were caught in the River Etne (Fig. 1) in the autumn of 2014. These fish were stripped in the local hatchery and gametes transferred to Matre Research station (IMR) (Fig. 1) where they were fertilised on the same day. Individual admixture rates, i.e., the proportion of the wild ancestry, was estimated for all parents using molecular markers^65^. This was done to verify that our wild salmon were not escapees from farms, F1 hybrids or backcrosses.

The commercial Mowi strain is the oldest strain of salmon used in commercial salmon aquaculture^23^. It was established in 1969, when large multi-sea winter fish were collected from the River Bolstad in the Vosso watercourse and the River Årøy, in addition to wild salmon caught in the sea outside of Western Norway, near Osterfjord and Sotra^30,66^. Offspring of salmon that had undergone 10 generations of selection were used as parents in this study. These fish had been reared at the Matre Research station (Institute of Marine Research) for one generation (from the egg stage in 2010), in the absence for directional selection. Upon reaching maturation in 2014, individuals were randomly selected as parents based on their family background (to avoid crossing siblings). Tissue samples of all parental salmon were collected to perform DNA parental assignment of offspring included in the study.

### Experimental rearing and treatments

All 15 families were created on November 20, 2014, at IMR. Eggs were incubated in the dark in separate family units until the eyed stage (average ambient water temperature: 5.4 °C, range 3.6–7.2 °C). After 398-degree days, eyed-eggs were on February 2, 2015, transported to the NINA Research Station at Ims in Norway (58°59′N, 5°58′E) (Fig. 1), for further incubation (average water temperature: 2.7 °C, range 0.9–8.5 °C). On April 21, 2015, after 612 degree days of incubation in separate family units, two weeks prior to the expected onset of exogenous feeding based on the developmental models of Crisp^67,68^, fry from each family were sorted out and mixed into replicated experimental units. Twenty yolk-sac fry per family were at this stage terminated and sampled for initial body mass. Thereafter, the mixed-family groups of domesticated, wild and hybrid salmon were immediately transferred to three experimental environments; (i) standard hatchery conditions, (ii) semi-natural stream conditions with intra-specific competition and (iii) semi-natural stream conditions with intra- and inter-specific competition as well as predation from larger sized brown trout (*Salmo trutta* L.).

For environment (i) 20 individuals per family (300 salmon in total) were sorted into replicated units and reared under standard hatchery conditions in two 1 m^2^ tanks (biomass density: 42.3 g m^−2^). These fish were kept under 24-hour light regime throughout the experiment and a commercial pelleted diet was provided ad libitum. While the overall aim of the study was to compare growth and survival of fry in the two semi-natural stream environments, the hatchery group was included as a control in order to compare overall fitness of the three genetic groups. Thus, ensuring that the experimental fish included in this study was representative to domesticated and wild fish used in other comparative studies performed under standard hatchery conditions^26,69^.

For environment (ii) 40 individuals per family (600 salmon fry in total) were sorted into replicates and planted in two enclosed semi-natural stream channels of semi-circular shape (30 m^2^, biomass density: 2.9 g m^−2^) with gravel substrate. These outdoor stream channels had a water level of 30–40 cm, a water discharge of 1.8–2.2 L s^−1^ and natural gravel substrate. Yolk-sac fry were carefully transferred to four artificial redds (10 salmon/family/redd) to allow for natural emergence. Only natural food items were available for the fish throughout the experiment, either from the gravel substratum or entering though the water inlet. However, one of the two replicates (No predation 2), received more sunlight than the other, resulting in varying levels of fouling of the gravel substratum between the two replicates.

For environment (iii) 40 individuals per family (600 salmon fry in total, biomass density 2.9 g m^−2^) was sorted into replicates units and planted into two semi-natural stream channels identical to those described above. Salmon fry were allowed to acclimatize to the new environment for five days before four 1-year-old brown trout (length 19.0 ± 1.4 cm, weight: 87.4 ± 23.2 g; mean ± SD and biomass density: 11.7 g m^−2^), were released into each of the two semi-natural stream channels on April 27, 2015. These brown trout originated from Fossbekk, located within the nearby Imsa watercourse, and had been reared at Ims Research station for two generations without any intentional selection. The gravel substratum provided the salmon fry with natural shelter to hide, while artificial hiding places for the brown trout were created by larger sized rocks being added to the artificial streams, prior to the initiation of the experiment. Again, one of the two replicates (Predation 1) received more sunlight than the other, resulting in varying levels of fouling of the gravel substratum between the two replicates.

All treatments were terminated approximately after six weeks, on June 8–11, 2015. The average ambient water temperatures throughout the experimental period was 9.8 °C, range 7.3–13.7 °C. Salmon fry was re-captured by electrofishing each of the four stream channels four times. All eight trout were re-captured in the same manner. All fry in the indoor tanks were sampled using dip-nets. All fish were euthanised by an overdose of BenzoakVet (www.europharma.no), prior to weight and length measurements. The individual’s specific growth rate (SGR) was calculated as SGR = (ln (final weight in grams) − ln (initial weight (family average in gram) × 100/t (in days). The caudal fin was sampled from all salmon fry and stored in ethanol for later DNA analysis back to family of origin. The stomach content of all trout was sampled, and later Sanger sequenced in order to detect if they recently had digested salmon fry.

### Classification

DNA was isolated from parental and offspring tissue samples. Tissue samples and six polymorphic microsatellite loci were genotyped on an ABI3730XL sequencer. Genotypes were identified using GeneMapper V4.0., and offspring assigned to family by the use of FAP v3.6^70^. See^26^ for details of the genotyping procedure. A total of three individuals were eventually excluded from the study due to poor DNA quality (one individual from the semi-natural environment without predation and two individuals from the control treatment).

DNA isolated from stomach content of all trout were sequenced using standard Sanger sequencing procedure on a 3730 DNA Analyzer (Thermo Fisher Scientific) using COI fish specific primers. The procedure was conducted following the description in^71^, modified from^72^. The eight sequences from the eight stomachs were manually checked, cleaned and BLASTED through Geneius 8.1.5, (default settings). Positive detection of salmon DNA in the trout stomach confirmed predation (see Supplementary File 1).

### Statistical analyses

All statistical analyses were performed in R version 3.6.1^73^.

Effects of genetic origin upon size as yolk-sac fry was investigated using a linear mixed effect model (gaussian distribution), fitted using the lmer function in the lme4 package^74^, where family, maternal identity and paternal identity were included as random intercept factors.

Effects of environment, genetic origin and size as yolk-sac fry on 1) growth (SGR) and 2) survival were investigated using a linear mixed effect model (gaussian distribution) and a generalized linear mixed effect model (binomial distribution), respectively. The models were fitted using the *lmer* function in the lme4 package^74^, and the full models also included the interaction between the fixed effects environment and genetic origin. Family, maternal identity, paternal identity and environmental replicate were included as random intercept factors in the full models. For the linear mixed effect growth model, model selection of both fixed and random effects were performed using the step function from the lmerTest package^75^. For the generalized liner mixed effect survival model, model selection of the random effects were performed by profiled confidence intervals visualized using the lattice package^76^, while model selection of the fixed effects, was performed using the drop1 function. The selected growth-model included all initial fixed effects; environment (3 factor levels), genetic origin (3 factor levels) and mean family size as yolk-sac fry (centered continuous variable), as well as the interaction between environment and genetic origin. The model also included the random effects of maternal identity (10 factor levels), paternal identity (10 factor levels) and replicate (6 factor levels). The selected survival-model included all initial fixed effects, as well as the interaction between environment and genetic origin. The model also included the random effects of family (15 factor levels), maternal identity and replicate.

Pair wise comparisons of the genetic groups within each treatment was performed by computing and contrasting estimated marginal means (least square means) using the emmeans function in the emmeans package^77^, which performs a Tukey-adjusted P-value correction, thus controlling for multiple comparisons.

### Ethics statement

The experimental protocol (permit number 7206) was approved by the Norwegian Animal Research Authority (NARA) on January 13, 2015. Welfare and use of experimental animals were performed in strict accordance with the Norwegian Animal Welfare Act of 19^th^ of June 2009, enforced on the 1^st^ of January 2010.

## Supplementary information

Supplementary file 1.

Dataset

## Data Availability

The data used in this study is available as Supplementary Information.
