# Supplementary material for: Domestication leads to increased predation susceptibility
Source: Sci Rep. 2020 Feb 6;10:1929. doi: 10.1038/s41598-020-58661-9 (PMC7005312; doi:10.1038/s41598-020-58661-9)
Supplement: Supplementary file 1 [file 41598_2020_58661_MOESM1_ESM.pdf]

Domestication leads to increased predation susceptibility

Monica F. Solberg[1*], Grethe Robertsen[2], Line E. Sundt-Hansen[2], Kjetil Hindar[2] and Kevin A. Glover[1,3]

[1] Institute of Marine Research, P.O. Box 1870 Nordnes, NO-5817 Bergen, Norway.

[2] Norwegian Institute for Nature Research (NINA), P.O. Box 5685 Torgarden, NO-7485 Trondheim, Norway.

[3] Department of Biology, University of Bergen, Norway

*correspondence to monica.solberg@hi.no

Supplementary files:

1. Family design.

2. Sequences of trout (individual Quant_ims_pred_T_2991- 2998) and their stomach content in fasta format.

3. Blast results documenting salmon DNA in a trout stomach (Quant_ims_pred_T_2997).

1) Family design

Ims - Family experimental crosses autumn 2014 (in Matre)

Family	Dam	Dam_Family	Wild/farmed	Sire	Sire_Family	Wild/farmed	Stock	Date established	Comments
IMS 1	2161	3	Farmed	E368	17	Farmed	Mowi	20.11.2015	Not included due to unsure health status of dam 2161
IMS 2	2161	3	Farmed	ETNE 11	NA	Wild	Hybrid	20.11.2015	Not included due to unsure health status of dam 2161
IMS 3	32CB	7	Farmed	1183	13	Farmed	Mowi	20.11.2015	
IMS 4	32CB	7	Farmed	ETNE 7	NA	Wild	Hybrid	20.11.2015	
IMS 5	2009	11	Farmed	0CE3	9	Farmed	Mowi	20.11.2015	
IMS 6	2009	11	Farmed	ETNE 8	NA	Wild	Hybrid	20.11.2015	
IMS 7	0E87	15	Farmed	2B75	5	Farmed	Mowi	20.11.2015	
IMS 8	0E87	15	Farmed	ETNE 9	NA	Wild	Hybrid	20.11.2015	
IMS 9	156A	17	Farmed	1936	19	Farmed	Mowi	20.11.2015	
IMS 10	156A	17	Farmed	ETNE 10	NA	Wild	Hybrid	20.11.2015	
IMS 11	321D	19	Farmed	EE05	1	Farmed	Mowi	20.11.2015	
IMS 12	321D	19	Farmed	ETNE 12	NA	Wild	Hybrid	20.11.2015	
IMS 13	ETNE 1	NA	Wild	ETNE 11	NA	Wild	Etne	20.11.2015	Not included to make a balanced design (mated with dam 2161).
IMS 14	ETNE 2	NA	Wild	ETNE 7	NA	Wild	Etne	20.11.2015	
IMS 15	ETNE 3	NA	Wild	ETNE 8	NA	Wild	Etne	20.11.2015	
IMS 16	ETNE 4	NA	Wild	ETNE 9	NA	Wild	Etne	20.11.2015	
IMS 17	ETNE 5	NA	Wild	ETNE 10	NA	Wild	Etne	20.11.2015	
IMS 18	ETNE 6	NA	Wild	ETNE 12	NA	Wild	Etne	20.11.2015	

2. Sequences of trout (individual Quant_ims_pred_T_2991- 2998) and their stomach content.

>Quant_ims_pred_T_2991_H09_033.ab1
TAGTATTTGGTGCCTGAGCCGGGATAGTCGGCACCGCCCTAAGTCTCTTGATTCGGGCAGAACTCAGCCAACC
CGGCGCCCTCCTAGGGGATGACCAGATTTATAACGTAATTGTTACAGCCCATGCCTTCGTAATAATTTTCTTTA
TAGTCATACCAATTATGATCGGCGGCTTTGGAAACTGATTAATCCCTCTCATAATCGGAGCCCCCGACATAGCA
TTCCCCCGAATGAATAACATAAGCTTCTGACTCCTCCCTCCGTCCTTTCTTCTCCTCCTAGCCTCGTCTGGAGTTG
AAGCCGGCGCTGGCACAGGATGAACAGTCTACCCCCCTCTAGCCGGCAATCTTGCCCACGCAGGAGCTTCCGT
TGACTTAACTATTTTCTCCCTCCATTTAGCTGGTATTTCCTCAATTTTGGGGGCCATTAATTTTATTACGACCATT
ATTAACATAAAACCCCCAGCCATCTCCCAATATCAAACTCCACTTTTTGTTTGGGCCGTGTTAGTCACCGCCGTC
CTCTTATTACTCTCCCTCCCTGTTTTAGCAGCAGGCATTACTATGCTACTCACAGACCGAAATCTTAATACCACTT
TCTTTGACCCGGCAGGCGGAGGGGATCCAATCTTATACCAACACCTCTTTTG

>Quant_ims_pred_T_2992_G09_034.ab1
GTATTTGGTGCCTGAGCCGGGATAGTCGGCACCGCCCTAAGTCTCTTGATTCGGGCAGAACTCAGCCAACCCG
GCGCCCTCCTAGGGGATGACCAGATTTATAACGTAATTGTTACAGCCCATGCCTTCGTAATAATTTTCTTTATA
GTCATACCAATTATGATCGGCGGCTTTGGAAACTGATTAATCCCTCTCATAATCGGAGCCCCCGACATAGCATT
CCCCCGAATGAATAACATAAGCTTCTGACTCCTCCCTCCGTCCTTTCTTCTCCTCCTAGCCTCGTCTGGAGTTGA
AGCCGGCGCTGGCACAGGATGAACAGTCTACCCCCCTCTAGCCGGCAATCTTGCCCACGCAGGAGCTTCCGTT
GACTTAACTATTTTCTCCCTCCATTTAGCTGGTATTTCCTCAATTTTGGGGGCCATTAATTTTATTACGACCATTA
TTAACATAAAACCCCCAGCCATCTCCCAATATCAAACTCCACTTTTTGTTTGGGCCGTGTTAGTCACCGCCGTCC
TCTTATTACTCTCCCTCCCTGTTTTAGCAGCAGGCATTACTATGCTACTCACAGACCGAAATCTTAATACCACTTT
CTTTGACCCGGCAGGCGGAGGGGATCCAATCTTATACCAACACCTCTTT

>Quant_ims_pred_T_2993_F09_035.ab1
TGCCTGAGCCGGGATAGTCGGCACCGCCCTAAGTCTCTTGATTCGGGCAGAACTCAGCCAACCCGGCGCCCTC
CTAGGGGATGACCAGATTTATAACGTAATTGTTACAGCCCATGCCTTCGTAATAATTTTCTTTATAGTCATACCA
ATTATGATCGGCGGCTTTGGAAACTGATTAATCCCTCTCATAATCGGAGCCCCCGACATAGCATTCCCCCGAAT
GAATAACATAAGCTTCTGACTCCTCCCTCCGTCCTTTCTTCTCCTCCTAGCCTCGTCTGGAGTTGAAGCCGGCGC
TGGCACAGGATGAACAGTCTACCCCCCTCTAGCCGGCAATCTTGCCCACGCAGGAGCTTCCGTTGACTTAACT
ATTTTCTCCCTCCATTTAGCTGGTATTTCCTCAATTTTGGGGGCCATTAATTTTATTACGACCATTATTAACATAA
AACCCCCAGCCATCTCCCAATATCAAACTCCACTTTTTGTTTGGGCCGTGTTAGTCACCGCCGTCCTCTTATTAC
TCTCCCTCCCTGTTTTAGCAGCAGGCATTACTATGCTACTCACAGACCGAAATCTTAATACCACTTTCTTTGACC
CGGCAGGCGGAGGGGATCCAATCTTATACCAACACCTCTTTTG

>Quant_ims_pred_T_2994_E09_036.ab1
ATTTGGTGCCTGAGCCGGGATAGTCGGCACCGCCCTAAGTCTCTTGATTCGGGCAGAACTCAGCCAACCCGGC
GCCCTCCTAGGGGATGACCAGATTTATAACGTAATTGTTACAGCCCATGCCTTCGTAATAATTTTCTTTATAGTC
ATACCAATTATGATCGGCGGCTTTGGAAACTGATTAATCCCTCTCATAATCGGAGCCCCCGACATAGCATTCCC
CCGAATGAATAACATAAGCTTCTGACTCCTCCCTCCGTCCTTTCTTCTCCTCCTAGCCTCGTCTGGAGTTGAAGC
CGGCGCTGGCACAGGATGAACAGTCTACCCCCCTCTAGCCGGCAATCTTGCCCACGCAGGAGCTTCCGTTGAC
TTAACTATTTTCTCCCTCCATTTAGCTGGTATTTCCTCAATTTTGGGGGCCATTAATTTTATTACGACCATTATTA
ACATAAAACCCCCAGCCATCTCCCAATATCAAACTCCACTTTTTGTTTGGGCCGTGTTAGTCACCGCCGTCCTCT
TATTACTCTCCCTCCCTGTTTTAGCAGCAGGCATTACTATGCTACTCACAGACCGAAATCTTAATACCACTTTCTT
TGACCCGGCAGGCGGAGGGGATCCAATCTTATACCAACACCTCTTTTG

>Quant_ims_pred_T_2995_D09_037.ab1
ATTTGGTGCCTGAGCCGGGATAGTCGGCACCGCCCTAAGTCTCTTGATTCGGGCAGAACTCAGCCAACCCGGC
GCCCTCCTAGGGGATGACCAGATTTATAACGTAATTGTTACAGCCCATGCCTTCGTAATAATTTTCTTTATAGTC
ATACCAATTATGATCGGCGGCTTTGGAAACTGATTAATCCCTCTCATAATCGGAGCCCCCGACATAGCATTCCC
CCGAATGAATAACATAAGCTTCTGACTCCTCCCTCCGTCCTTTCTTCTCCTCCTAGCCTCGTCTGGAGTTGAAGC
CGGCGCTGGCACAGGATGAACAGTCTACCCCCCTCTAGCCGGCAATCTTGCCCACGCAGGAGCTTCCGTTGAC
TTAACTATTTTCTCCCTCCATTTAGCTGGTATTTCCTCAATTTTGGGGGCCATTAATTTTATTACGACCATTATTA
ACATAAAACCCCCAGCCATCTCCCAATATCAAACTCCACTTTTTGTTTGGGCCGTGTTAGTCACCGCCGTCCTCT
TATTACTCTCCCTCCCTGTTTTAGCAGCAGGCATTACTATGCTACTCACAGACCGAAATCTTAATACCACTTTCTT
TGACCCGGCAGGCGGAGGGGATCCAATCTTATACCAACACCTCTTTTG

>Quant_ims_pred_T_2996_C09_038.ab1
ATAGTCATACCAATTATGATCGGCGGCTTTGGAAACTGATTAATCCCTCTCATAATCGGAGCCCCCGACATAGC
ATTCCCCCGAATGAATAACATAAGCTTCTGACTCCTCCCTCCGTCCTTTCTTCTCCTCCTAGCCTCGTCTGGAGTT
GAAGCCGGCGCTGGCACAGGATGAACAGTCTACCCCCCTCTAGCCGGCAATCTTGCCCACGCAGGAGCTTCC
GTTGACTTAACTATTTTCTCCCTCCATTTAGCTGGTATTTCCTCAATTTTGGGGGCCATTAATTTTATTACGACCA
TTATTAACATAAAACCCCCAGCCATCTCCCAATATCAAACTCCACTTTTTGTTTGGGCCGTGTTAGTCACCGCCG
TCCTCTTATTACTCTCCCTCCCTGTTTTAGCAGCAGGCATTACTATGCTACTCACAGACCGAAATCTTAATACCA
CTTTCTTTGACCCGGCAGGCGGANGGGATCCAATCTTATACCAACACCTCTTTTG

>Quant_ims_pred_T_2997_B09_039.ab1
NNNNNNNNNNNNNNNNTNNGTATTTGGTGCCTGAGCCGGNATAGTCGGCACCGCCCTAAGTCTCTTGATTCG
NGNAGAACTCAGNCNNCCNGGCGCCNTNCNGGGGGATGACCAAATTTATAACGTAATTGTTACAGCCCATG
CCTTCGTNATAATTTTCTTTATAGTCATACCNATTATGATCGGCGGCTTTGGAAACTGATTAATNCCTCTNATAA
TCGGNGCCCCCGACATAGCATTCCCCCGAATGAATAACATANNNTTNTGACTNCNCCCTCCNTCCTTTCTTCTC
CTCCTGGCCTCNTCTGGAGTTGAAGCCGGCGCTGGCACCGGATGAACAGTCTACCCCCCTCTAGNNNGNAAT
CTTGCCCACGCAGGAGCTTCCGTTGACTTAACTATTTTNNCCCTCCATTNNNNTGGTATTTCCTTCAATNNNNN
NGGCCATTAATTTTTATTACNACCATNATTAANATAAAACCCCCAGCNTATCTCTCANTATCAAACTCCCACTTT
TTGNTTGGANCTGNGNTTAGTCACTGCCGTCCTCTTNNGNNACTCTCCCTCCCTGTTTCTAGCAGCAGGCATT
ACTNNNNACTACTNNACAGANCGAAATCTAAATACCACTTTTCTTTGACCNGGGCAGGNGNAGNANNNCCC
AATCTTAGNACCAACNNNNCTTTNGNNNNNNCGGCCNNCCANNANTNNANNCNNNNNNNNNNNN
NNNNNNNNNNNNNNNNN

>Quant_ims_pred_T_2998_A09_040.ab1
NNNNNNNNNNNNNNNNTTTAGTATTTGGTGCCTGAGCCGGGATAGTCGGCACCGCCCTAAGTCTCTTGATTC
GGGCAGAACTCAGCCAACCCGGCGCCCTCCTAGGGGATGACCAGATTTATAACGTAATTGTTACAGCCCATGC
CTTCGTAATAATTTTCTTTATAGTCATACCAATTATGATCGGCGGCTTTGGAAACTGATTAATCCCTCTCATAAT
CGGAGCCCCCGACATAGCATTCCCCCGAATGAATAACATAAGCTTCTGACTCCTCCCTCCGTCCTTTCTTCTCCT
CCTAGCCTCGTCTGGAGTTGAAGCCGGCGCTGGCACAGGATGAACAGTCTACCCCCCTCTAGCCGGCAATCTT
GCCCACGCAGGAGCTTCCGTTGACTTAACTATTTTCTCCCTCCATTTAGCTGGTATTTCCTCAATTTTGGGGGCC
ATTAATTTTATTACGACCATTATTAACATAAAACCCCCAGCCATCTCCCAATATCAAACTCCACTTTTTGTTTGGG
CCGTGTTAGTCACCGCCGTCCTCTTATTACTCTCCCTCCCTGTTTTAGCAGCAGGCATTACTATGCTACTCACAG
ACCGAAATCTTAATACCACTTTCTTTGACCCGGCAGGCGGANGGGANCCAATCTTATACCAACACCTCTTTTGN
TTCTTNGGCCACCCANAANTNNANNNNNNNNNTTTTTNNNNNNNNNNNNNGN

3. Blast results documenting salmon DNA in a trout stomach (Quant_ims_pred_T_2997).

>MN245140 Salmo salar voucher KLMMEA201925 cytochrome oxidase subunit 1 (COI) g
ene, partial cds; mitochondrial Length = 624 E-value = 3.01e-
172, Score = 333, Bitscore = 616.055, Identities = 355/373 (95%), Positives = 369/373 (98%), Gaps =
0/373 (0%) Frame = +1
Quant_ims_pred_T_2997_B09_039.ab1   1 ATAGTCGGCACCGCCCTAAGTCTCTTGATTCGAGCAGAACT
CAGCCAACCNGGCGCCCTN 60   ATAGTCGGCACCGCCCTAAGTCTCTTGATTCGAG
CAGAACTCAGCCA CC+GGCGCCCT+   MN245140   4 ATAGTCGGCACCGCCCTAAGTCTC
TTGATTCGAGCAGAACTCAGCCAGCCTGGCGCCCTT 63   Quant_ims_pred_T_2997_B09_039.ab1   61
CTGGGGGATGACCAAATTTATAACGTAATTGTTACAGCCCATGCCTTCGTNATAATTTTC 120
CTGGG GATGACCAAATTTATAACGTAATTGTTACAGCCCATGCCTTCGT+ATAATTTTC   MN2451
40   64 CTGGGAGATGACCAAATTTATAACGTAATTGTTACAGCCCATGCCTTCGTCATAATTT
TC 123   Quant_ims_pred_T_2997_B09_039.ab1   121 TTTATAGTCATACCNATTATGATCGGCGGCTTTG
GAAACTGATTAATNCCTCTNATAATC 180   TTTATAGTCATACC+ATTATGATCGGCG
GCTTTGGAAACTGATTAAT+CCTCT+ATAATC   MN245140   124 TTTATAGTCATACCGATT
ATGATCGGCGGCTTTGGAAACTGATTAATTCCTCTTATAATC 183   Quant_ims_pred_T_2997_B09_039.a
b1   181 GGNGCCCCCGACATAGCATTCCCCCGAATGAATAACATAATNTTNTGACTNCTCCCTCCN 240
GG+GCCCCCGACATAGCATTCCCCCGAATGAATAACATAA +TT+TGACT+CTCCCTCC+
MN245140   184 GGGGCCCCCGACATAGCATTCCCCCGAATGAATAACATAAGTTTTTGACTT
CTCCCTCCC 243   Quant_ims_pred_T_2997_B09_039.ab1   241 TCCTTTCTTCTCCTCCTGGCCTCNTCTG
GAGTTGAAGCCGGCGCTGGCACCGGATGAACA 300   TCCTTTCTTCTCCTCCTGGCC
TC+TCTGGAGTTGAAGCCGGCGCTGGCACCGGATGAACA   MN245140   244 TCCTTTCTT
CTCCTCCTGGCCTCATCTGGAGTTGAAGCCGGCGCTGGCACCGGATGAACA 303   Quant_ims_pred_T_299
7_B09_039.ab1   301 GTCTACCCCCCTCTAGCNGGNAATCTTGCCCACGCAGGAGCTTCCGTTGACTTAACTA
TT 360   GTCTACCCCCCTCTAGC+GG+AATCTTGCCCACGCAGGAGCTTCCGTTGACT
TAACTATT   MN245140   304 GTCTACCCCCCTCTAGCAGGTAATCTTGCCCACGCAGGAGC
TTCCGTTGACTTAACTATT 363   Quant_ims_pred_T_2997_B09_039.ab1   361 TTCTCCCTCCATT 373
TT TCCCTCCATT   MN245140   364 TTTTCCCTCCATT 376

Salmo salar voucher KLMMEA201925 cytochrome oxidase subunit 1 (COI) gene, partial cds;
mitochondrial

1 10 20 30 40 50

| | | | | |

ATAGTCGGCACCGCCCTAAGTCTCTTGATTCGAGCAGAACTCAGCCAGCC

TGGCGCCCTTCTGGGAGATGACCAAATTTATAACGTAATTGTTACAGCCC

ATGCCTTCGTCATAATTTTCTTTATAGTCATACCGATTATGATCGGCGGC

TTTGGAAACTGATTAATTCCTCTTATAATCGGGGCCCCCGACATAGCATT

CCCCCGAATGAATAACATAAGTTTTTGACTTCTCCCTCCCTCCTTTCTTC

TCCTCCTGGCCTCATCTGGAGTTGAAGCCGGCGCTGGCACCGGATGAACA

GTCTACCCCCCTCTAGCAGGTAATCTTGCCCACGCAGGAGCTTCCGTTGA

CTTAACTATTTTTTCCCTCCATT

>MN245140 Salmo salar voucher KLMMEA201925 cytochrome oxidase subunit 1 (COI) g
ene, partial cds; mitochondrial Length = 624 E-value = 3.01e-
172, Score = 333, Bitscore = 616.055, Identities = 355/373 (95%), Positives = 369/373 (98%), Gaps = 0/373 (0%) Frame = +1
Quant_ims_pred_T_2997_B09_039.ab1   1 ATAGTCGGCACCGCCCTAAGTCTCTTGATTCGAGCAGAACT CAGCCAACCNGGCGCCCTN 60   ATAGTCGGCACCGCCCTAAGTCTCTTGATTCGAG CAGAACTCAGCCA CC+GGCGCCCT+   MN245140   4 ATAGTCGGCACCGCCCTAAGTCTC TTGATTCGAGCAGAACTCAGCCAGCCTGGCGCCCTT 63   Quant_ims_pred_T_2997_B09_039.ab1   61 CTGGGGGATGACCAAATTTATAACGTAATTGTTACAGCCCATGCCTTCGTNATAATTTTC 120
CTGGG GATGACCAAATTTATAACGTAATTGTTACAGCCCATGCCTTCGT+ATAATTTTC   MN2451 40   64 CTGGGAGATGACCAAATTTATAACGTAATTGTTACAGCCCATGCCTTCGTCATAATTT TC 123   Quant_ims_pred_T_2997_B09_039.ab1   121 TTTATAGTCATACCNATTATGATCGGCGGCTTTG GAAACTGATTAATNCCTCTNATAATC 180   TTTATAGTCATACC+ATTATGATCGGCG GCTTTGGAAACTGATTAAT+CCTCT+ATAATC   MN245140   124 TTTATAGTCATACCGATT ATGATCGGCGGCTTTGGAAACTGATTAATTCCTCTTATAATC 183   Quant_ims_pred_T_2997_B09_039.a b1   181 GGNGCCCCCGACATAGCATTCCCCCGAATGAATAACATAATNTTNTGACTNCTCCCTCCN 240
GG+GCCCCCGACATAGCATTCCCCCGAATGAATAACATAA +TT+TGACT+CTCCCTCC+
MN245140   184 GGGGCCCCCGACATAGCATTCCCCCGAATGAATAACATAAGTTTTTGACTT CTCCCTCCC 243   Quant_ims_pred_T_2997_B09_039.ab1   241 TCCTTTCTTCTCCTCCTGGCCTCNTCTG GAGTTGAAGCCGGCGCTGGCACCGGATGAACA 300   TCCTTTCTTCTCCTCCTGGCC TC+TCTGGAGTTGAAGCCGGCGCTGGCACCGGATGAACA   MN245140   244 TCCTTTCTT CTCCTCCTGGCCTCATCTGGAGTTGAAGCCGGCGCTGGCACCGGATGAACA 303   Quant_ims_pred_T_299 7_B09_039.ab1   301 GTCTACCCCCCTCTAGCNGGNAATCTTGCCCACGCAGGAGCTTCCGTTGACTTAACTA TT 360   GTCTACCCCCCTCTAGC+GG+AATCTTGCCCACGCAGGAGCTTCCGTTGACT TAACTATT   MN245140   304 GTCTACCCCCCTCTAGCAGGTAATCTTGCCCACGCAGGAGC TTCCGTTGACTTAACTATT 363   Quant_ims_pred_T_2997_B09_039.ab1   361 TTCTCCCTCCATT 373
TT TCCCTCCATT   MN245140   364 TTTTCCCTCCATT 376

Salmo salar voucher KLMMEA201925 cytochrome oxidase subunit 1 (COI) gene, partial cds; mitochondrial

```
1      10      20      30      40      50
|      |       |       |       |       |
ATAGTCGGCACCGCCCTAAGTCTCTTGATTCGAGCAGAACTCAGCCAGCC
TGGCGCCCTTCTGGGAGATGACCAAATTTATAACGTAATTGTTACAGCCC
ATGCCTTCGTCATAATTTTCTTTATAGTCATACCGATTATGATCGGCGGC
TTTGGAAACTGATTAATTCCTCTTATAATCGGGGCCCCCGACATAGCATT
CCCCCGAATGAATAACATAAGTTTTTGACTTCTCCCTCCCTCCTTTCTTC
TCCTCCTGGCCTCATCTGGAGTTGAAGCCGGCGCTGGCACCGGATGAACA
GTCTACCCCCCTCTAGCAGGTAATCTTGCCCACGCAGGAGCTTCCGTTGA
CTTAACTATTTTTTCCCTCCATT
```

>MN245139 Salmo salar voucher KLMMEA201924 cytochrome oxidase subunit 1 (COI) gene, partial cds; mitochondrial Length = 652 E-value = 3.01e-172, Score = 333, Bitscore = 616.055, Identities = 355/373 (95%), Positives = 369/373 (98%), Gaps = 0/373 (0%) Frame = +1
Quant_ims_pred_T_2997_B09_039.ab1 1 ATAGTCGGCACCGCCCTAAGTCTCTTGATTCGAGCAGAACTCAGCCAACCNGGCGCCCTN 60 ATAGTCGGCACCGCCCTAAGTCTCTTGATTCGAGCAGAACTCAGCCA CC+GGCGCCCT+ MN245139 32 ATAGTCGGCACCGCCCTAAGTCTCTTGATTCGAGCAGAACTCAGCCAGCCTGGCGCCCTT 91 Quant_ims_pred_T_2997_B09_039.ab1 61 CTGGGGGATGACCAAATTTATAACGTAATTGTTACAGCCCATGCCTTCGTNATAATTTTC 120 CTGGG GATGACCAAATTTATAACGTAATTGTTACAGCCCATGCCTTCGT+ATAATTTTC MN245139 92 CTGGGAGATGACCAAATTTATAACGTAATTGTTACAGCCCATGCCTTCGTCATAATTTTC 151 Quant_ims_pred_T_2997_B09_039.ab1 121 TTTATAGTCATACCNATTATGATCGGCGGCTTTGGAAACTGATTAATNCCTCTNATAATC 180 TTTATAGTCATACC+ATTATGATCGGCGGCTTTGGAAACTGATTAAT+CCTCT+ATAATC MN245139 152 TTTATAGTCATACCGATTATGATCGGCGGCTTTGGAAACTGATTAATTCCTCTTATAATC 211 Quant_ims_pred_T_2997_B09_039.ab1 181 GGNGCCCCCGACATAGCATTCCCCCGAATGAATAACATAATNTTNTGACTNCTCCCTCCN 240 GG+GCCCCCGACATAGCATTCCCCCGAATGAATAACATAA +TT+TGACT+CTCCCTCC+ MN245139 212 GGGGCCCCCGACATAGCATTCCCCCGAATGAATAACATAAGTTTTTGACTTCTCCCTCCC 271 Quant_ims_pred_T_2997_B09_039.ab1 241 TCCTTTCTTCTCCTCCTGGCCTCNTCTGGAGTTGAAGCCGGCGCTGGCACCGGATGAACA 300 TCCTTTCTTCTCCTCCTGGCCTC+TCTGGAGTTGAAGCCGGCGCTGGCACCGGATGAACA MN245139 272 TCCTTTCTTCTCCTCCTGGCCTCATCTGGAGTTGAAGCCGGCGCTGGCACCGGATGAACA 331 Quant_ims_pred_T_2997_B09_039.ab1 301 GTCTACCCCCCTCTAGCNGGNAATCTTGCCCACGCAGGAGCTTCCGTTGACTTAACTATT 360 GTCTACCCCCCTCTAGC+GG+AATCTTGCCCACGCAGGAGCTTCCGTTGACTTAACTATT MN245139 332 GTCTACCCCCCTCTAGCAGGTAATCTTGCCCACGCAGGAGCTTCCGTTGACTTAACTATT 391 Quant_ims_pred_T_2997_B09_039.ab1 361 TTCTCCCTCCATT 373 TT TCCCTCCATT MN245139 392 TTTTCCCTCCATT 404

Salmo salar voucher KLMMEA201924 cytochrome oxidase subunit 1 (COI) gene, partial cds; mitochondrial

```
1      10      20      30      40      50
|      |       |       |       |       |
ATAGTCGGCACCGCCCTAAGTCTCTTGATTCGAGCAGAACTCAGCCAGCC
TGGCGCCCTTCTGGGAGATGACCAAATTTATAACGTAATTGTTACAGCCC
ATGCCTTCGTCATAATTTTCTTTATAGTCATACCGATTATGATCGGCGGC
TTTGGAAACTGATTAATTCCTCTTATAATCGGGGCCCCCGACATAGCATT
CCCCCGAATGAATAACATAAGTTTTTGACTTCTCCCTCCCTCCTTTCTTC
TCCTCCTGGCCTCATCTGGAGTTGAAGCCGGCGCTGGCACCGGATGAACA
GTCTACCCCCCTCTAGCAGGTAATCTTGCCCACGCAGGAGCTTCCGTTGA
CTTAACTATTTTTTCCCTCCATT
```

>MN245138 Salmo salar voucher KLMMEA201923 cytochrome oxidase subunit 1 (COI) gene, partial cds; mitochondrial Length = 652 E-value = 3.01e-172, Score = 333, Bitscore = 616.055, Identities = 355/373 (95%), Positives = 369/373 (98%), Gaps = 0/373 (0%) Frame = +1
Quant_ims_pred_T_2997_B09_039.ab1 1 ATAGTCGGCACCGCCCTAAGTCTCTTGATTCGAGCAGAACTCAGCCAACCNGGCGCCCTN 60 ATAGTCGGCACCGCCCTAAGTCTCTTGATTCGAGCAGAACTCAGCCA CC+GGCGCCCT+ MN245138 32 ATAGTCGGCACCGCCCTAAGTCTCTTGATTCGAGCAGAACTCAGCCAGCCTGGCGCCCTT 91 Quant_ims_pred_T_2997_B09_039.ab1 61 CTGGGGGATGACCAAATTTATAACGTAATTGTTACAGCCCATGCCTTCGTNATAATTTTC 120 CTGGG GATGACCAAATTTATAACGTAATTGTTACAGCCCATGCCTTCGT+ATAATTTTC MN245138 92 CTGGGAGATGACCAAATTTATAACGTAATTGTTACAGCCCATGCCTTCGTCATAATTTTC 151 Quant_ims_pred_T_2997_B09_039.ab1 121 TTTATAGTCATACCNATTATGATCGGCGGCTTTGGAAACTGATTAATNCCTCTNATAATC 180 TTTATAGTCATACC+ATTATGATCGGCGGCTTTGGAAACTGATTAAT+CCTCT+ATAATC MN245138 152 TTTATAGTCATACCGATTATGATCGGCGGCTTTGGAAACTGATTAATTCCTCTTATAATC 211 Quant_ims_pred_T_2997_B09_039.ab1 181 GGNGCCCCCGACATAGCATTCCCCCGAATGAATAACATAATNTTNTGACTNCTCCCTCCN 240 GG+GCCCCCGACATAGCATTCCCCCGAATGAATAACATAA +TT+TGACT+CTCCCTCC+ MN245138 212 GGGGCCCCCGACATAGCATTCCCCCGAATGAATAACATAAGTTTTTGACTTCTCCCTCCC 271 Quant_ims_pred_T_2997_B09_039.ab1 241 TCCTTTCTTCTCCTCCTGGCCTCNTCTGGAGTTGAAGCCGGCGCTGGCACCGGATGAACA 300 TCCTTTCTTCTCCTCCTGGCCTC+TCTGGAGTTGAAGCCGGCGCTGGCACCGGATGAACA MN245138 272 TCCTTTCTTCTCCTCCTGGCCTCATCTGGAGTTGAAGCCGGCGCTGGCACCGGATGAACA 331 Quant_ims_pred_T_2997_B09_039.ab1 301 GTCTACCCCCCTCTAGCNGGNAATCTTGCCCACGCAGGAGCTTCCGTTGACTTAACTATT 360 GTCTACCCCCCTCTAGC+GG+AATCTTGCCCACGCAGGAGCTTCCGTTGACTTAACTATT MN245138 332 GTCTACCCCCCTCTAGCAGGTAATCTTGCCCACGCAGGAGCTTCCGTTGACTTAACTATT 391 Quant_ims_pred_T_2997_B09_039.ab1 361 TTCTCCCTCCATT 373 TT TCCCTCCATT MN245138 392 TTTTCCCTCCATT 404

Salmo salar voucher KLMMEA201923 cytochrome oxidase subunit 1 (COI) gene, partial cds; mitochondrial

```
1      10      20      30      40      50
|      |       |       |       |       |
ATAGTCGGCACCGCCCTAAGTCTCTTGATTCGAGCAGAACTCAGCCAGCC
TGGCGCCCTTCTGGGAGATGACCAAATTTATAACGTAATTGTTACAGCCC
ATGCCTTCGTCATAATTTTCTTTATAGTCATACCGATTATGATCGGCGGC
TTTGGAAACTGATTAATTCCTCTTATAATCGGGGCCCCCGACATAGCATT
CCCCCGAATGAATAACATAAGTTTTTGACTTCTCCCTCCCTCCTTTCTTC
TCCTCCTGGCCTCATCTGGAGTTGAAGCCGGCGCTGGCACCGGATGAACA
GTCTACCCCCCTCTAGCAGGTAATCTTGCCCACGCAGGAGCTTCCGTTGA
CTTAACTATTTTTTCCCTCCATT
```

>MN245134 Salmo salar voucher KLMMEA201922 cytochrome oxidase subunit 1 (COI) g
ene, partial cds; mitochondrial Length = 652 E-value = 3.01e-
172, Score = 333, Bitscore = 616.055, Identities = 355/373 (95%), Positives = 369/373 (98%), Gaps =
0/373 (0%) Frame = +1
Quant_ims_pred_T_2997_B09_039.ab1 1 ATAGTCGGCACCGCCCTAAGTCTCTTGATTCGAGCAGAACT
CAGCCAACCNGGCGCCCTN 60 ATAGTCGGCACCGCCCTAAGTCTCTTGATTCGAG
CAGAACTCAGCCA CC+GGCGCCCT+ MN245134 32 ATAGTCGGCACCGCCCTAAGTCT
CTTGATTCGAGCAGAACTCAGCCAGCCTGGCGCCCTT 91 Quant_ims_pred_T_2997_B09_039.ab1 61
CTGGGGGATGACCAAATTTATAACGTAATTGTTACAGCCCATGCCTTCGTNATAATTTTC 120
CTGGG GATGACCAAATTTATAACGTAATTGTTACAGCCCATGCCTTCGT+ATAATTTTC MN2451
34 92 CTGGGAGATGACCAAATTTATAACGTAATTGTTACAGCCCATGCCTTCGTCATAATTT
TC 151 Quant_ims_pred_T_2997_B09_039.ab1 121 TTTATAGTCATACCNATTATGATCGGCGGCTTTG
GAAACTGATTAATNCCTCTNATAATC 180 TTTATAGTCATACC+ATTATGATCGGCG
GCTTTGGAAACTGATTAAT+CCTCT+ATAATC MN245134 152 TTTATAGTCATACCGATT
ATGATCGGCGGCTTTGGAAACTGATTAATTCCTCTTATAATC 211 Quant_ims_pred_T_2997_B09_039.a
b1 181 GGNGCCCCCGACATAGCATTCCCCCGAATGAATAACATAATNTTNTGACTNCTCCCTCCN 240
GG+GCCCCCGACATAGCATTCCCCCGAATGAATAACATAA +TT+TGACT+CTCCCTCC+
MN245134 212 GGGGCCCCCGACATAGCATTCCCCCGAATGAATAACATAAGTTTTTGACTT
CTCCCTCCC 271 Quant_ims_pred_T_2997_B09_039.ab1 241 TCCTTTCTTCTCCTCCTGGCCTCNTCTG
GAGTTGAAGCCGGCGCTGGCACCGGATGAACA 300 TCCTTTCTTCTCCTCCTGGCC
TC+TCTGGAGTTGAAGCCGGCGCTGGCACCGGATGAACA MN245134 272 TCCTTTCTT
CTCCTCCTGGCCTCATCTGGAGTTGAAGCCGGCGCTGGCACCGGATGAACA 331 Quant_ims_pred_T_299
7_B09_039.ab1 301 GTCTACCCCCCTCTAGCNGGNAATCTTGCCCACGCAGGAGCTTCCGTTGACTTAACTA
TT 360 GTCTACCCCCCTCTAGC+GG+AATCTTGCCCACGCAGGAGCTTCCGTTGACT
TAACTATT MN245134 332 GTCTACCCCCCTCTAGCAGGTAATCTTGCCCACGCAGGAGC
TTCCGTTGACTTAACTATT 391 Quant_ims_pred_T_2997_B09_039.ab1 361 TTCTCCCTCCATT 373
TT TCCCTCCATT MN245134 392 TTTTCCCTCCATT 404

Salmo salar voucher KLMMEA201922 cytochrome oxidase subunit 1 (COI) gene, partial cds; mitochondrial
